# Supplementary material for: Variations in Diacron-Reactive Oxygen Metabolites and Biological Antioxidant Potential Across Reproductive Phases and Parities in Sows Reared Under Different Production Systems
Source: Animals (Basel). 2025 Sep 9;15(18):2638. doi: 10.3390/ani15182638 (PMC12466595; doi:10.3390/ani15182638)

animals

MDPI

Supplementary Table S1. Composition of dietary ingredients during the gestation period

Ingredient	Detail	Proportion(%)
Cereals	Corn, Powder, Rice, Wheat flour	58
Bran	Wheat bran, Rice bran	17
Vegetable oilseeds	Rapeseed oil cake, Soybean oil cake	12
Animal oilseeds	Fish meal, Chicken meal	3
Other	Alfalfa meal, Calcium carbonate, Salt, Feed yeast, Silica, Confectionery meal, Calcium phosphate	10

Composition table	Proportion(%)
Crude protein	15
Crude fat	2
Crude fiber	10
Ash	10
Ca	0.75
P	0.6
Total digestible nutrients	70

Supplementary Table S2. Composition of dietary ingredients during the lactation pe-

Ingredient	Detail	Proportion(%)
Cereals	Corn, Rice,	63
Vegetable oilseeds	Rapeseed oil cake, Soybean oil cake	27
Animal oilseeds	Fish meal, Chicken meal	3
Bran	Rice bran	1
Other	Alfalfa meal, Calcium carbonate, Salt, Feed yeast, Silica, Confectionery meal, Calcium phosphate	6

Composition table	Proportion(%)
Crude protein	18
Crude fat	3.5
Crude fiber	10
Ash	10
Ca	0.75
P	0.5
Total digestible nutrients	78.5

Supplement Figure S1. The study timeline included three blood sampling points.

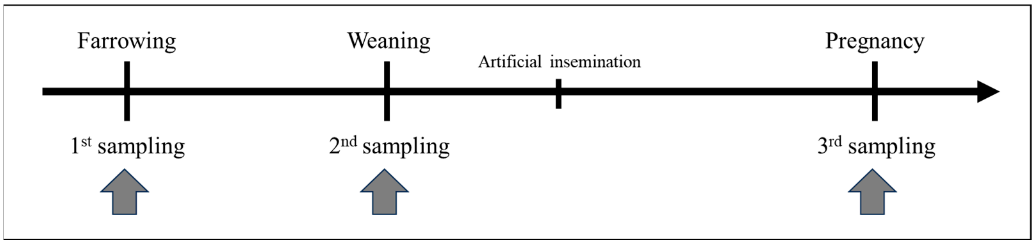

Supplement: Supplementary file 1 [file animals-15-02638-s001.zip › animals-3761373-supplementary.pdf]
